# Wheat EST resources for functional genomics of abiotic stress

**DOI:** 10.1186/1471-2164-7-149

**Published:** 2006-06-13

**Authors:** Mario Houde, Mahdi Belcaid, François Ouellet, Jean Danyluk, Antonio F Monroy, Ani Dryanova, Patrick Gulick, Anne Bergeron, André Laroche, Matthew G Links, Luke MacCarthy, William L Crosby, Fathey Sarhan

**Affiliations:** 1 Département des Sciences biologiques, Université du Québec à Montréal, C.P. 8888, Succ. Centre-ville, Montréal QC, H3C 3P8, Canada; 2 Département d'Informatique, Université du Québec à Montréal, C.P. 8888, Succ. Centre-ville, Montréal QC, H3C 3P8, Canada; 3 Biology Department, Concordia University, 7141 Sherbrooke Street West, Montreal QC, H4B 1R6, Canada; 4 Agriculture et Agroalimentaire Canada, Centre de recherches de Lethbridge, 5403, 1st Avenue South, C.P. 3000, Lethbridge AB, T1J 4B1, Canada; 5 Department of Biological Sciences, University of Windsor, 401 Sunset ave, Windsor ON, N9B 3P4, Canada; 6 Department of Computer Science, University of Saskatchewan, 176 Thorvaldson Building, 110 Science Place, Saskatoon SK, S7N 5C9, Canada

## Abstract

**Background:**

Wheat is an excellent species to study freezing tolerance and other abiotic stresses. However, the sequence of the wheat genome has not been completely characterized due to its complexity and large size. To circumvent this obstacle and identify genes involved in cold acclimation and associated stresses, a large scale EST sequencing approach was undertaken by the Functional Genomics of Abiotic Stress (FGAS) project.

**Results:**

We generated 73,521 quality-filtered ESTs from eleven cDNA libraries constructed from wheat plants exposed to various abiotic stresses and at different developmental stages. In addition, 196,041 ESTs for which tracefiles were available from the National Science Foundation wheat EST sequencing program and DuPont were also quality-filtered and used in the analysis. Clustering of the combined ESTs with d2_cluster and TGICL yielded a few large clusters containing several thousand ESTs that were refractory to routine clustering techniques. To resolve this problem, the sequence proximity and "bridges" were identified by an e-value distance graph to manually break clusters into smaller groups. Assembly of the resolved ESTs generated a 75,488 unique sequence set (31,580 contigs and 43,908 singletons/singlets). Digital expression analyses indicated that the FGAS dataset is enriched in stress-regulated genes compared to the other public datasets. Over 43% of the unique sequence set was annotated and classified into functional categories according to Gene Ontology.

**Conclusion:**

We have annotated 29,556 different sequences, an almost 5-fold increase in annotated sequences compared to the available wheat public databases. Digital expression analysis combined with gene annotation helped in the identification of several pathways associated with abiotic stress. The genomic resources and knowledge developed by this project will contribute to a better understanding of the different mechanisms that govern stress tolerance in wheat and other cereals.

## Background

Cold acclimation (CA) allows hardy plants to develop the efficient freezing tolerance (FT) mechanisms needed for winter survival. During the period of exposure to low temperature (LT), numerous biochemical, physiological and metabolic functions are altered in plants, and these changes are regulated by LT mostly at the gene expression level. The identification of LT-responsive genes is therefore required to understand the molecular basis of CA. Cold-induced genes and their products have been isolated and characterized in many species. In wheat and other cereals, the expression of several genes during cold acclimation was found to be positively correlated with the capacity of each genotype and tissue to develop FT [1]. Furthermore, abiotic stresses that have a dehydrative component (such as cold, drought and salinity) share some responses. It is therefore expected that, in addition to the genes regulated specifically by each stress, some genes will be regulated by multiple stresses. The availability of wheat genotypes with varying degree of FT makes this species an excellent model to study freezing tolerance and other abiotic stresses. The identification of new genes involved in the cold response will provide invaluable tools to further our understanding of the metabolic pathways of cold acclimation and the acquisition of superior freezing tolerance of hardy genotypes.

Major genomics initiatives have generated valuable data for the elucidation of the expressed portion of the genomes of higher plants. The genome sequencing of *Arabidopsis thaliana *was completed in 2000 [2] while the finished sequence for rice was recently published [3]. The relatively small genome size of these model organisms was a key element in their selection as the first plant genomes to be sequenced with extensive coverage. On the other hand, the allohexaploid wheat genome is one of the largest among crop species with a haploid size of 16.7 billion bp [4], which is 110 and 40 times larger than *Arabidopsis *and rice respectively [5]. The large size, combined with the high percentage (over 80%) of repetitive non-coding DNA, presents a major challenge for comprehensive sequencing of the wheat genome. However, a significant insight into the expressed portion of the wheat genome can be gained through large-scale generation and analysis of ESTs. cDNA libraries prepared from different tissues exposed to various stress conditions and developmental stages are valuable tools to obtain the expressed and stress-regulated portion of the genome. This approach was used in several species such as oat [6], barley [7], tomato [8] and poplar [9]. The sequencing of cDNAs gives direct information on the mature transcripts for the coding portion of the genome that can subsequently be used for gene identification and functional studies. The availability of wheat genomics data in the public datasets has grown rapidly through major initiatives [10,11]. However, additional ESTs are needed to complete the identification of the expressed genes under different growth conditions and from different genotypes. This will contribute to a more complete representation of the genome through identification of new genes and extension of contigs for the majority of genes that have incomplete sequence coverage. Towards this goal, the Functional Genomics of Abiotic Stress (FGAS) program initiated an EST sequencing effort directed toward the study of abiotic stress, with an emphasis on cold acclimation [12]. To increase gene diversity in the EST population and increase the probability of identifying those associated with freezing tolerance, different cDNA libraries were prepared from winter wheat tissues exposed for various times to low temperature, together with select libraries derived from tissues exposed to other stresses or at different developmental stages. In this report, we describe the generation of 73,521 high quality ESTs from wheat stress-associated cDNA libraries. In order to perform the assembly and digital expression analyses, these ESTs were supplemented with wheat ESTs for which sequence quality data was available. These include the NSF [13] and DuPont datasets, which will be referred to as the 'NSF-DuPont' dataset in this report. Digital expression analyses identified a large number of genes that were associated with cold acclimation and other stresses. Expression analyses and functional classification provided important information about the different metabolic and regulatory pathways that are possibly associated with cellular adjustment to environmental stresses. These new EST resources are an important addition to publicly available resources especially in relation to the study of abiotic stresses in cereals.

## Results and discussion

The large-scale FGAS wheat EST sequencing project was undertaken to identify new genes associated with abiotic stress and to provide physical resources for functional studies. We have developed a unique wheat EST resource from eleven cDNA libraries prepared from tissues at different developmental stages and exposed to different stress conditions (Table 1). The EST collections from FGAS, NSF and DuPont were analyzed and classified into functional categories.

### Assembly and identification of new wheat genes

We have used EST sequences and quality values from the corresponding tracefiles of large datasets (FGAS, NSF and DuPont) to assemble 75,488 different wheat sequences (31,580 contigs, 36,388 singletons and 7,520 singlets). Among these datasets, the FGAS project produced 11,225 unique sequences (2,824 contigs, 6,663 singletons and 1,738 singlets) indicating that the FGAS ESTs encompass a large subset of unique transcripts. These sequences were analyzed using BLASTN on the db_est database and filtered for wheat sequences with two different cut-off e-values to identify new wheat genes. With an e-25 cut-off value, we found that 2,304 genes had no homologous wheat ESTs (Table 2). After filtering these genes against the wheat protein database with TBLASTX, there were still 2,243 proteins showing no homology to known proteins. With an e-05 cut-off, 1,581 genes had no homologs in wheat. After filtering these against the protein database, 1,470 non-homologous sequences remained. These unique wheat sequences were then BLASTed against *Arabidopsis*, rice, and finally nr db EST (Table 2). In *Arabidopsis*, we found that only 5 of the remaining FGAS wheat sequences had a strong (e-25) similarity using BLASTN while 253 of the remaining sequences had homologs when filtered with the *Arabidopsis *protein database (count down to 1,985). A similar trend was found in *Arabidopsis *using a lower sequence similarity cut-off (e-05). The remaining unique gene count was reduced by several hundred after comparing protein homologs in rice (counts down to 1674 at e-25 and down to 855 at e-05) demonstrating that several genes common between rice and wheat are absent in *Arabidopsis *(Table 2). The remaining unique ESTs were BLASTed against the non redundant database to determine whether homologs were present in other organisms. At an e-05, there were 795 ESTs showing no significant similarity to known domains in genes from other species. It is possible that some of these genes derive from unknown micro-organisms contaminating the plant tissues, and/or from residual genomic DNA in the RNA samples used for cDNA synthesis. However, the majority of these sequences have ORFs encoding proteins larger than 30 amino acids, with an average predicted protein size of over 100 amino acids. This suggests that the unidentified genes do represent novel wheat genes.

The Institute for Genomic research (TIGR) wheat gene index (Release 10.0) shows that only 6,431 of the 44,954 wheat contigs (14%) were successfully allocated a known Molecular Function using Gene Ontology, compared to the classification done for *Arabidopsis *in which 12,558 of the 28,900 contigs (42%) have a known Molecular Function. Therefore, prior to this report, *Arabidopsis *had almost twice as many genes annotated with at least one defined function compared to wheat (12,558 vs 6,431). The classification of the complete dataset (FGAS and NSF-DuPont datasets) allowed the tentative annotation of 43.3% of the genes. As expected, most of the annotated sequences were in contigs (57.6%) while the percentage of annotated singletons/singlets was much lower (30.8%). We have thus been able to functionally annotate 29,556 different sequences, an almost 5-fold increase in annotated sequences compared to TIGR. This is a significant contribution that broadens the available wheat public annotation dataset for downstream functional studies. These results demonstrate that a large number of wheat genes are poorly characterized and stress the fact that major efforts in functional analyses are needed.

### Enrichment for stress-regulated genes in the FGAS dataset

**Figure 1:**
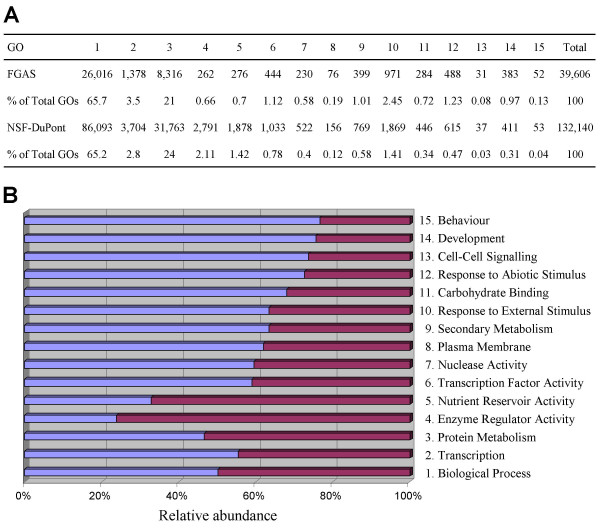
**Abundance of annotated ESTs in FGAS contigs relative to NSF-DuPont contigs within select GO classes**. **A) **Number of annotated ESTs. The GO counts were added for each dataset and the percentage of ESTs for each GO was calculated based on this total count. **B) **The relative abundance for each GO is compared between the FGAS (blue) and the NSF-DuPont (red) datasets by comparing the percentage of each GO as determined in A. GO categories: 1. Biological Process GO:0008150; 2. Transcription GO:0006350; 3. Protein Metabolism GO:0019538; 4. Enzyme Regulator Activity GO:0030234; 5. Nutrient Reservoir Activity GO:0045735; 6. Transcription Factor Activity GO:0003700; 7. Nuclease Activity GO:0004518; 8. Plasma Membrane GO:0005886; 9. Secondary Metabolism GO:0019748; 10. Response to External Stimulus GO:0009605; 11. Carbohydrate Binding GO:0030246; 12. Response to Abiotic Stimulus GO:0009628; 13. Cell-Cell Signalling GO:0007267; 14. Development GO:0007275; 15. Behaviour GO:0007610.

Comparative analysis of the FGAS ESTs and NSF-DuPont ESTs based on Gene Ontology (GOslim) showed that several GO classes are more represented in FGAS than in the NSF-DuPont dataset (Figure 1). When general GO classes are compared (GOs 1 to 3; Biological Process, Transcription and Protein Metabolism), no major differences in the number of ESTs were found. Similarly, most GOslim classes showed less than 25% difference between the two datasets. However, GOs 4 and 5 (Enzyme Regulator Activity and Nutrient Reservoir Activity) had a lower representation while GOs 6 to 15 (Transcription Factor Activity, Nuclease Activity, Plasma Membrane, Secondary Metabolism, Response to External Stimulus, Carbohydrate Binding, Response to Abiotic Stimulus, Cell-Cell Signalling, Development and Behavior) were more abundant in the FGAS dataset (Figure 1).

To identify genes that are differentially represented between the two datasets, the relative abundance of ESTs was analyzed and referred to as digital expression analysis. For each contig, the number of ESTs from FGAS (excluding ESTs derived from Suppressive Subtractive Hybridization; SSH) was divided by the number of ESTs from NSF-DuPont and the ratio was normalized to correct for the difference in size between the two datasets (54,032 non SSH EST sequences for the FGAS dataset and 196,041 sequences for the NSF-DuPont dataset). Thus, after normalization, the relative expression level for a contig having 1 EST from each dataset would result in a relative expression of 3.62X in FGAS compared to NSF-DuPont (a ratio of 1 multiplied by 196,041/54,032). Since the SSH technique aims to enrich differentially expressed cDNAs, the ESTs derived from the SSH libraries were analysed separately to avoid a bias in the number of ESTs in a contig, which could invalidate the digital expression analysis approach.

The data indicated that over 75% of the contigs have ratios that vary by less than two-fold, suggesting a similar representation of ESTs between the FGAS (less SSH) and the NSF-DuPont datasets. The remaining 25% of contigs showed more than two-fold difference in abundance (Table 3; see additional file 1: Table1.xls) in the FGAS dataset. When 5- and 10-fold ratios are used as cut-off, 6.6% and 1.7% of the contigs are retained respectively. Most of the differences are due to genes that are over-represented in the FGAS dataset (for the 5-fold cut-off, 1959 genes are over- and 136 genes are under-represented, see Table 3). With a higher cut-off (20-fold differential abundance), only 61 contigs are over expressed and 5 are under-expressed. An analysis of these highly over-represented contigs showed that a good proportion (52%) of these show homology to genes that were previously reported to be over-expressed under stress (see references in Table 4). This high percentage of positive identification suggests that the NSF-DuPont collection was a good reference dataset for digital expression analysis of the FGAS dataset.

Our digital expression analysis relies on the presence of ESTs from both datasets in a same contig (since we cannot divide by 0). We have also identified 542 contigs that contained at least 3 ESTs from FGAS but none from the NSF-DuPont dataset (See additional file 2: Table 2.xls). Table 5 lists the 90 genes that contain at least 5 ESTs unique to FGAS, and many of these are similar to genes that have previously been reported to be over-expressed under stress. Although the unique contigs in the FGAS dataset may represent transcripts that are specific to the cultivar used in our study, there is a possibility that they may represent novel genes that are induced by environmental stress.

In *Arabidopsis*, microarray experiments have shown that about 10% of the genes are over-or under-expressed by at least two-fold upon exposure to cold acclimation conditions [14]. Based on our previous northern and microarray analyses, we have estimated that the same proportion of wheat genes is cold-regulated (Sarhan *et al*., unpublished results). If we consider a conservative estimate of 30,000 wheat genes (90,000 if we consider the A, B and D genomes), this means that around 3,000 genes would be cold-regulated. A similar number of genes was identified when we used a 5-fold cut-off differential expression (2,095 differentially expressed contigs, Table 3) and added the 542 contigs having at least 3 ESTs that are unique to the FGAS dataset. Using these criteria, our analyses resulted in a total of 2,637 contigs or 8.4% of the contigs generated in our assembly (31,580 contigs). Considering that 95% of the EST sequences were derived from libraries constructed from cold-acclimated plants, these genes represent candidate genes likely regulated by low temperature and other stresses. However, many of these may be differentially expressed as a consequence of the temperature shift and metabolic adjustment and might not be involved in conferring or regulating increased tolerance to stress. It would be of interest to analyse these 2,637 genes to identify those relevant to LT tolerance and other stresses in cereals. To verify the conservation of the stress response between wheat and *Arabidopsis*, we first identified the *Arabidopsis *proteins having homology (e-25) to the 2,637 wheat proteins identified in our study, using the TAIR protein database. The homology search resulted in the identification of 1,551 *Arabidopsis *proteins. Most of the genes encoding these proteins are represented on the Affymetrix and MWG microarrays. This allowed us to obtain their expression profiles from the available public data [14,15]. Our analysis indicated that 941 genes are cold-regulated and 890 are drought-regulated (See additional file 1: Table 1.xls and additional file 2: Table 2.xls). There are 678 genes regulated by both stresses, with a total of 1153 different *Arabidopsis *genes that are stress-regulated. Therefore, there are over 44% of the 2,637 putative wheat stress-regulated genes that have a homolog regulated by stress in *Arabidopsis*, suggesting overlapping responses between the two species.

As a complementary approach to identifying new wheat genes that may be differentially expressed, different SSH libraries were produced to identify genes over-expressed after brief (1 day) or long (21–49 days) periods of cold acclimation. Different cultivars that may help to identify other components of freezing tolerance such as pathogen resistance to snow molds were used for these analyses. A total of 3,873 contigs containing 18,610 SSH ESTs were obtained with 2,969 contigs (76.7%) tentatively annotated. Unique contigs from SSH libraries are potentially a good source to mine for new genes associated with cold acclimation. Overall, 225 contigs unique to the SSH libraries (See additional file 3: Table 3.xls) were identified, among which 74 were annotated (Table 6). We found that 11 of the 74 annotated SSH contigs (or 15% of the unique SSH contigs) have corresponding genes (high similarity based on BLASTX e-values) that are over-expressed more than 5-fold in the differentially-expressed FGAS contigs. These results suggest that unique SSH contigs contain candidate genes that could be involved in abiotic stress tolerance.

### Metabolic pathways associated with differentially expressed genes

**Figure 2:**
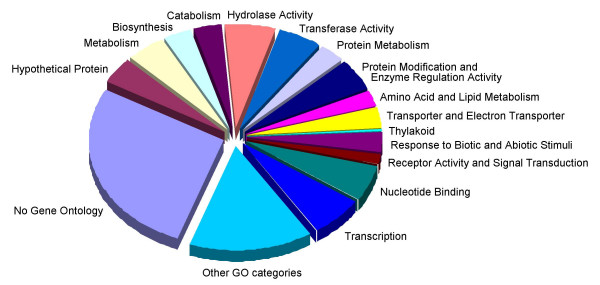
**Functional classification of FGAS contigs containing ESTs that are over or under-represented more than 5-fold, or that contain more than 3 unique ESTs**. The contigs belonging to the following GO terms were used: GO0008152 Metabolism; GO0009058 Biosynthesis; GO0009056 Catabolism; GO0016787 Hydrolase Activity; GO0016740 Transferase Activity; GO0019538 Protein Metabolism; GO0006464 and GO0030234 Protein Modification and Enzyme Regulator Activity; GO0006519 and GO0006629 Amino Acid and Lipid Metabolism; GO0005215 and GO0005489 Transporter and Electron Transporter Activity; GO0009579 Thylakoid; GO0009607 and GO 0009628 Response to Biotic and Abiotic Stimulus; GO0004872 and GO0007165 Receptor Activity and Signal Transduction; GO000166 Nucleotide Binding; Transcription Factors only from GO0006350 and GO0003677 (other DNA Binding Proteins were transferred to "Other GO categories"); a class was made for the mention "Hypothetical Protein" and for the mention "No Gene Ontology" while the "Other GO Categories" regroups several GO terms with small number of contigs.

GO slim annotation was used to subdivide the 2,637 stress-regulated genes into function categories to gain insight into their putative role during cold acclimation and abiotic stresses. The results show that a large proportion of these contigs were annotated under a limited number of GO classes (Figure 2). Over 53.7% of the contigs were grouped into 14 GO categories while 27.5% of the contigs were designated "No Gene Ontology" and 4.2% were classified as "Hypothetical Protein", a term used to designate open reading frames predicted from the *Arabidopsis *or rice genomic DNA. The remaining contigs with other GO categories were grouped together in one category (14.6%).

A plethora of physiological and metabolic adjustments occur during cold acclimation and in response to other stresses. The regulation of genes involved in temperature, drought and salt stresses is known to reflect the cross-talk between different signalling pathways [16]. However, few studies have identified multiple genes that are stress-regulated and that belong to a same metabolic pathway. Our analyses enabled us to position several genes in their respective metabolic pathway, suggesting that these pathways are involved in stress responses. Since it is beyond the scope of this report to cover all possible pathways involved, we highlight some of the key elements that likely contribute to the stress response and tolerance. Unless specifically indicated, all enzymes discussed are encoded by transcripts that are over-represented by at least 5-fold in the FGAS dataset.

### Amino acid metabolism

Genes encoding proteins involved in primary metabolism pathways have been identified in the contigs with an over-representation of FGAS ESTs and cover several aspects of plant metabolic adjustments. Amino acid metabolism and the TCA cycle are the major pathways that generate precursors for various biological molecules. ESTs encoding several enzymes that are involved in the synthesis of arginine, cysteine, lysine, methionine, serine, phenylalanine, proline and tryptophan are over-represented by more than 5-fold. These amino acids are precursors for the synthesis of several specialized metabolites. Two contigs encode the enzyme delta-1-pyrroline-5-carboxylate synthetase that is involved in proline biosynthesis, a metabolite that was found to increase during cold acclimation and drought stress [17]. Similarly, two contigs encode glutamate decarboxylase (GAD1), which is involved in the synthesis of gamma-aminobutyric acid (GABA), a non protein amino acid known to accumulate during cold acclimation and proposed to function in oxidative stress tolerance [18]. Several contigs encode enzymes involved in the metabolism of cysteine, an important precursor of glutathione involved in the modulation of oxidative stress. These include two different cysteine synthases and a putative O-acetylserine (thiol) synthase (OASTL). Over-expression of different isoforms of OASTL can increase thiol content in different transgenic plants and increase tolerance to abiotic stress such as exposure to elevated levels of cadmium [19].

### Lipid metabolism

**Figure 3:**
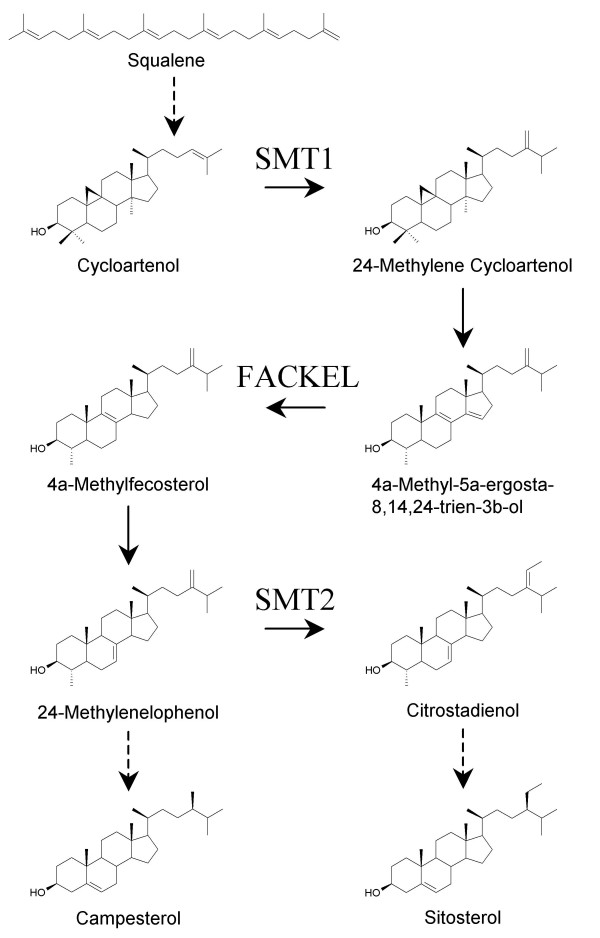
**Plant sterols pathway**. ESTs encoding several enzymes of the sterol pathways are over-represented in the FGAS dataset. Three enzymes are involved in the production of squalene from which cycloarthenol is obtained. The FACKLE and SMT2 enzymes are involved in the production of sitosterol with a concomitant decrease in campesterol.

ESTs encoding different putative lipases and other proteins involved in lipid oxidation (acyl-CoA oxidase, MutT/nudix protein like, dihydrolipoamide acetyltransferase, b-keto acyl reductase, enoyl-ACP reductase, enoyl-CoA-hydratase, 3-hydroxyisobutyryl-coenzyme A hydrolase) are over-represented in the FGAS dataset while the acyl-carrier protein III involved in lipid synthesis is under-represented. These results suggest that lipid degradation occurs concomitantly with a reduction in the synthesis of short chain lipids. On the other hand, ESTs encoding enzymes involved in the synthesis of specialized lipids such as ATP citrate lyase α-subunit and the long chain fatty acid enzyme acetyl-CoA carboxylase are more abundant among FGAS ESTs. ESTs corresponding to several enzymes involved in sterol metabolism are also over-represented, suggesting major lipid modifications in membranes during cold acclimation. ESTs encoding three enzymes involved in the alternate pathway of isopentenyl pyrophosphate and squalene synthesis (1-deoxy-D-xylulose 5-phosphate reductoisomerase, 1-deoxy-D-xylulose-5-phosphate synthase, squalene synthase), three key enzymes of the sterol pathways (cycloartenol synthase, C14-sterol reductase (FACKEL), and 24-methylenelophenol methyltransferase) (Figure 3), and other enzymes such as sterol 4-alpha-methyl-oxidase, which can add to the variety of sterols produced, are also over-represented. The putative over-expression of several enzymes in the sterol pathway supports the previous observation of an increased production of membrane sterols [20]. These authors showed that the concentration of membrane sterols increases during cold acclimation and that this effect is more prominent in tolerant rye cultivars. Interestingly, sitosterol increases while campesterol decreases during acclimation, suggesting that the C24 methyltransferase that is putatively over-expressed in the FGAS dataset may be the SMT-2 transferase that diverts the methylenelophenol into the sitosterol pathway (see Figure 3; [21]). A search through the protein database has shown that the C24 methyltransferase has a much greater homology with SMT2 (7e-143) than with SMT1 (4e-63) supporting that the C24 methyltransferase is SMT2. The over-representation of FGAS ESTs in two contigs encoding stearoyl-acyl-carrier protein desaturase and two contigs encoding CDP-diacylglycerol synthase suggests that other important lipid modifying activities also occur in response to cold acclimation. Stearoyl-acyl-carrier protein desaturase is involved in the desaturation of existing lipids to form double bonds rendering the lipids more fluid at low temperature. This is an important adjustment associated with membrane stability at low temperature [20]. The over-expression of CDP-diacylglycerol synthase was previously shown to favour the synthesis of phosphatidylinositol [22]. In addition, one contig encodes a phosphoethanolamine N-methyltransferase. This enzyme is induced by low temperature and catalyzes the three sequential methylation steps to form phosphocholine, a key precursor of phosphatidylcholine and glycinebetaine in plants – metabolites known to be important in conferring tolerance to osmotic stresses such as low temperature, drought and salinity [23].

### Secondary metabolism

Several contigs encode key enzymes involved in the biosynthesis of secondary metabolites such as phenylalanine ammonia lyase, cinnamyl alcohol dehydrogenase, and caffeoyl-CoA O-methyltransferase. Several enzymes are involved in the synthesis of methionine and its derivatives. The digital expression data suggest that the S-adenosylmethionine (SAM) cycle becomes more active during stress since contigs encoding three major enzymes of the cycle (S-adenosylmethionine synthetase, methionine S-methyltransferase, and S-adenosylhomocysteine hydrolase) are over-represented in FGAS. This pathway can provide SAM, the precursor molecule needed for nicotianamine biosynthesis. Four different contigs encoding nicotianamine synthase or nicotianamine aminotransferase are over-represented in FGAS. These enzymes are involved in nicotianamine and phytosiderophores synthesis and were found to be induced under iron deficiency [24,25]. The SAM cycle also provides the one carbon precursor for the methylation steps required for methyltransferase activities. At least 20 different contigs encoding methyltransferases contain ESTs that are over-represented in FGAS.

### Transport activity

During cold acclimation, the cell mobilizes several transport systems to adapt to cold conditions. One of the major effects of extracellular freezing is the reduced apoplastic water pressure and the rapid flow of water from the intracellular compartment to the apoplasm. Some of the consequences include the need for water and ion regulation as well as protection against dehydration. Two different contigs encoding aquaporins are highly abundant in FGAS (a contig with 12 ESTs found only in the FGAS dataset and a contig with ESTs over-represented 18-fold). These proteins likely play an important role in the regulation of the outward water flow. Similarly, several contigs associated with transport of ions or other small solutes are more highly represented, such as anion/sugar transporters, major facilitator superfamily antiporters, MATE efflux family transporters, nitrate transporters, cation exchangers, calcium and zinc transporters, betaine/proline transporters, and amino acid transporters. These different transporters are potential regulators controlling the flow of ions and other solutes that become more concentrated as water is drawn out of the cell during freezing. An interesting transporter activity is the phosphatidylinositol-phosphatidylcholine transfer protein which can contribute to the turnover of these lipids in the membrane. This pathway is involved in the accumulation of the compatible solute betaine that was reported to increase tolerance to drought and freezing [26]. Another mechanism involved in cell protection against higher ionic content include the replacement of water with compatible solutes such glycerol, glucose, sorbitol, proline and betaine. ESTs encoding hydroquinone glucosyltransferase, an interesting enzyme responsible for the synthesis of arbutin, are over-represented over 7-fold in the FGAS dataset. Glycosylated hydroquinone is very abundant in freezing and desiccation tolerant plants. It was suggested to accumulate up to 100 mM in the resurrection plant *Myrothamnus flabellifolia *and to increase membrane stability of artificial liposomes and thylakoids, possibly through the insertion of the phenol moiety in the phospholipid bilayer [27]. These authors showed that the lipid membrane composition is an important element for the cryoprotective effect of arbutin. In support of this observation, several contigs with an over-representation of FGAS ESTs encoding transporters of compatible solutes and lipid modifying enzymes were identified.

### Proteins involved in cryoprotection

One strategy that hardy plants such as wheat use to tolerate subzero temperatures is the accumulation of freezing tolerance associated proteins such as antifreeze proteins (AFPs) and dehydrins [28]. AFPs exhibit two related activities *in vitro*. The first is to increase the difference between the freezing and melting temperatures of aqueous solutions, a property known as thermal hysteresis. The second is ice recrystallization inhibition (IRI), where the growth of large ice crystals is inhibited, thus reducing the possibility of physical damage within frozen tissues [29]. In winter wheat and rye, several AFPs similar to pathogenesis-related proteins such as chitinases, glucanases, thaumatins and ice recrystallization inhibition proteins were identified [30-32]. Many contigs encoding chitinases, β-1,3-glucanases and thaumatin-like proteins contain ESTs that are over-represented in FGAS. Hincha *et al*. [33] reported that different cryoprotective proteins were able to protect thylakoids from freezing injury *in vitro*. Wheat ice recrystallization inhibition proteins are partly homologous to, and were annotated as, phytosulfokine receptors and were present in several contigs containing ESTs over-expressed in FGAS.

The dehydrins are hydrophilic proteins resistant to heat denaturation composed largely of repeated amino acid sequence motifs. They possess regions capable of forming an amphipathic α-helix. These properties may enable them to protect cells against freezing damage by stabilizing proteins and membranes during conditions of dehydration [28]. The most studied dehydrins are the WCS120 family, the WCOR410 and the chloroplastic WCS19 dehydrins. Genes encoding these proteins are highly over-represented in the FGAS dataset (Table 4, Table 5, and see additional file 1: Table 1.xls).

### Photosynthesis

During cold acclimation, the chloroplast continues to receive as much light as at normal temperature but its thermal biochemical reactions are reduced. This results in an excess of light energy whereby electrons accumulate mostly in Q_A _[34]. The reduced capacity to transfer electrons through PSII requires metabolic adjustments on a short term basis through redox balance, and communication between the chloroplast and the nucleus to modify gene expression for adaptation on a longer term basis. Freezing tolerant plants were previously shown to better cope with photoinhibition than less tolerant cultivars [34]. Although the number of genes classified under the GO "Thylakoids" is only 13, the genes identified indicate that putative changes in expression occur for genes encoding components of both the photosystem I (PSI) and the photosystem II (PSII). Several studies have reported changes in PSII during cold acclimation [34], The D1 and D2 proteins were shown to be sensitive to excess energy and to turn over more rapidly at low temperature and high light [35]. ESTs encoding the D2 protein are over-expressed by 7.2-fold in FGAS suggesting that the PSII adapts to low temperature conditions. On the other hand, the transcript encoding PSII Z is less represented in FGAS. A reduced amount of this protein may lead to a reduction in active antennas and allow a reduction in electron flow towards the PSII. ESTs encoding two other proteins of the PSII complex are over-represented (29.8 kDa and 20 kDa protein). These proteins belong to the same PsbP protein family which has 4 members in *Arabidopsis*. Recent results using RNAi have shown that this lumen protein is both essential and quantitatively related to PSII efficiency and stability. This suggests that their over-expression could improve electron flow through PSII [36,37]. Another limiting factor in the electron flow is the availability of CO_2_. Several contigs with over-represented ESTs in the FGAS dataset encode carbonic anhydrase (carbonic anhydrase chloroplast precursor, dioscorin class A and nectarin III). This enzyme is known in C4 plants to concentrate CO_2 _at its site of fixation. In the C3 plant wheat, this enzyme was previously shown to be modulated by nitrogen deficiency to maintain optimal CO_2 _concentrations [38]. The over-expression of this enzyme could thus help to efficiently use the CO_2 _and available light energy at low temperature. Failure to dissipate excess light energy could lead to oxidative stress, which needs to be controlled. A contig encoding a putative serine hydroxymethyltransferase is over-represented in the FGAS dataset. Hydroxymethyltransferases play a critical role in controlling the cell damage caused by abiotic stresses such as high light and salt, supporting the notion that photorespiration forms part of the dissipatory mechanisms of plants to minimize production of reactive oxygen species (ROS) in the chloroplast and to mitigate oxidative damage [39].

Very few studies have documented the modulation of PSI under stress conditions. The excess light or low temperature can decrease stromal NADP/NADPH ratio and it has been proposed that the cytochrome b6f complex can be regulated by the stromal redox potential possibly via a thioredoxin mediated mechanism (see [40]). The PSI components are largely integrated and composed of many subunits making it energetically expensive for the cell to produce. It has been suggested that cells might modulate PSI activity by varying the amount of the small and mobile plastocyanin protein carrying the reducing power [41]. The over-representation of ESTs encoding this protein in FGAS (represented by 27 ESTs within contig CL187Contig5) suggests that this PSI electron relay component becomes more active during cold acclimation and may be important in relieving the pressure caused by electrons accumulating in Q_B_. The mobile plastocyanin molecule is a limiting factor in the electron transfer from PSII to PSI. The increased expression of plastocyanin may result in an increased activity of PSI under low temperature and may help freezing tolerant plants maintain their energy balance compared to less tolerant plants. We have previously shown that several proteins involved in improving photosynthesis, including plastocyanin, are expressed at low levels under low excitation pressure (20°C/50 μE) but markedly accumulate when transferred to 5°C under the same light regime [42]. A mutation in the PSI-E subunit was also shown to have a great impact on PSII as it becomes easily affected by photoinhibition even under low light [43]. Similarly mutants in the PSI-N subunit, which participates in the docking of PC, are impaired in PSI activity [44]. The over-representation of ESTs encoding the PSI-E and PSI-N subunits in the FGAS dataset could thus provide an integrated response to reduce photoinhibition. In order to maintain a proper NADP/NADPH ratio, the malate valve could be activated to transfer excess reducing power to the cytoplasm [45]. ESTs encoding two PSI components are less abundant in FGAS. One of these is a subunit of the chloroplastic NADH dehydrogenase equivalent to the mitochondrial enzyme. Interestingly, the *FRO1 *gene was recently shown to encode the mitochondrial NADH dehydrogenase counterpart which plays a role in controlling ROS and the ability of *Arabidopsis *to respond to low temperature [46]. An excess of ROS in mitochondria was proposed to affect the induction of CBF transcription factors and cold acclimation. The chloroplastic NADH dehydrogenase may also affect the ability to induce CBF if the ROS that accumulate during photoinhibition at low temperature are not detoxified. Tolerant plants may adapt their photosystems to avoid the accumulation of ROS in chloroplasts, thus allowing a strong CBF response and a stable induction of downstream cold-regulated genes. This hypothesis may explain why tolerant plants are able to maintain a strong expression of several freezing tolerance-associated genes while less tolerant plants show transient, reduced expression of these genes at low temperature [1].

### Signalling cascades and transcription factors

Among the contigs with an over-representation in FGAS ESTs, we identified several proteins involved in the synthesis or perception of different hormones. These include enzymes of the ethylene, auxin and jasmonic acid metabolism; brassinosteroid LRR receptor, receptor-like kinases CLAVATA2 and PERK1, and phytosulfokine receptor. Contigs encoding several proteins involved in signalling cascades were also found such as calcium binding proteins, diacylglycerol kinase, lipid phosphate phosphatase-2, inositol 1-monophosphatase, GTP-binding proteins, MAP kinases and MAPKK, serine/threonine kinase, CIPK-like protein-1, histidine kinase-2, and protein phosphatases 2A and 2C.

The potentially increased activity of the various signalling pathways is associated with a differential expression of many families of transcription factors (TF; Table 7). The results show that at least 220 contigs contain ESTs encoding TF that are over- or under-represented more than two-fold in the FGAS dataset. Using a more stringent cut-off excludes some TF that may not be strongly regulated, but should also reduce the number of false positives. With a 5-fold cut-off, 151 TF were identified, with 30 of them being contigs unique to FGAS. The most highly represented TF families are the zinc fingers, WRKY, AP2, Myb and NAC. Several members of these families were previously identified as being responsive to various stresses. The most studied members are those of the AP2 family, in particular the CBF/DREB subfamily. CBF members are involved in the cold/drought responses [47]. We have identified 3 different contigs, with a 5-fold over-representation in the FGAS dataset, that contain CBF-like binding factors and 5 unique FGAS contigs containing at least 3 ESTs (annotated as CBF-like, CBF1-like, CBF3-like, C-repeat binding factor 3-like, C-repeat/DRE binding factor 3, CRT/DRE binding factor 2, DRE binding factor-2). Expression profiling using qRT-PCR has confirmed that transcripts corresponding to 7 of the 8 contigs are over-expressed at specific time points during cold acclimation (Sarhan *et al*. unpublished results). Expression of the CBF genes in *Arabidopsis *was shown to be regulated by members of the bHLH family [48]. We have identified 7 contigs encoding bHLH members that are over-represented by two-fold, with two of them being over-represented more than 5-fold (Table 7). However, the genes encoding the bHLH ICE proteins in *Arabidopsis *are not cold-induced. Although the expression pattern with regards to cold inducibility of the ICE genes could be different between wheat and *Arabidopsis*, the isolation of the full length genes, phylogenetic analysis and expression studies are required to determine if any of the over-represented bHLH encode ICE homologs. In addition to the CBFs and bHLH families, several other TF families may be part of other stress components associated with abiotic stress such as drought, salinity, oxidative, etc. Interestingly, several genes that control flowering have also been identified (FLT, Gigantea, MADS, CO, Aintegumenta). These genes are most likely associated with the vernalization response in wheat as was recently shown for *TaVRT1 *and *TaVRT2 *[49,50].

## Conclusion

The large number of ESTs annotated from FGAS and NSF-DuPont datasets represents an important resource for the wheat community. Digital expression analyses of these datasets provide an overview of metabolic changes and specific pathways that are regulated under stress conditions in wheat and other cereals. The information generated will help construct network models of abiotic stress responses that will facilitate computational predictions and direct future experimental work like the development of models such as the "Metabolic pathways of the diseased potato" [51] or MapMan for the analysis of gene expression data in *Arabidopsis *[52]. The results could facilitate the understanding of cellular mechanisms involving groups of gene products that act in coordination in response to environmental stimuli.

## Methods

A total of eleven different cDNA libraries were prepared from hexaploid wheat (*Triticum aestivum*) for the FGAS EST sequencing project and are summarized in Table 1. Cultivar Norstar was used for Libraries 2 to 6 to represent various tissues, developmental stages and stress conditions. Six subtracted cDNA libraries (suppression subtractive hybridization; SSH), named TaLT2 to TaLT7, were also prepared from two different wheat lines (CI14106 and PI178383) and cv Norstar as a complementary approach to isolate differentially expressed transcripts. The "Library 1" and TaLT1 libraries were not used for the large scale EST sequencing FGAS project since the former was not prepared in a Gateway-compatible vector and the latter was generated to optimize the SSH protocol.

### Preparation of the cDNA libraries

#### Growth conditions

For Libraries 2 and 3, the seeds were germinated in water-saturated vermiculite for 7 days at 20°C and 70% relative humidity under an irradiance of 200 μmol m^-2 ^sec^-1 ^and a 15-hr photoperiod. At the end of this period, the aerial parts (crowns and leaves) and roots of control plants were sampled and individually frozen. Cold acclimation was performed by subjecting germinated seedlings to a temperature of 4°C with a 12-hr photoperiod for 1, 23 and 53 days under an irradiance of 200 μmol m^-2 ^sec^-1^. Seedlings were watered with a nutrient solution (0.5 g/l 20:20:20; N:P:K). Salt stress was induced by watering with the nutrient solution containing 200 mM NaCl for 0.5, 3 and 6-hr. Aerial parts of cold-acclimated plants were sampled for Library 2 and roots of both cold-acclimated and salt-stressed plants were sampled for Library 3.

For Library 4, two different water stress conditions were used. For bench experiments, seeds were germinated for 7 days as described for Library 2. At the end of this period, plants were removed from vermiculite and left at room temperature on the table without water for 1, 2, 3 and 4 days before sampling. For growth chamber experiments, seeds were germinated in a water-saturated potting mix (50% black earth and 50% ProMix) for 7 days under an irradiance of 200 μmol m^-2 ^sec^-1^. The temperature was maintained at 20°C with a 15-hr photoperiod under a relative humidity of 70%. After this period, watering of plants was stopped. Four time points were sampled during a two weeks period; the first after wilting was observed and the last, two weeks later, and consisted of living crown and stem tissues (leaf tissue was yellow and thus not included in the sampled material).

For Library 5, seeds were germinated for 7 days and cold-treated for 49 days (full vernalization) as described for Library 2. Seedlings were then potted in water-saturated potting mix and transferred to flower inducing conditions (20°C and a 15-hr photoperiod). Tissues were sampled as follows: 1 cm crown sections after 30 days of cold treatment; 1 cm vernalized (49-day cold-treated) crown sections that were exposed to flower inducing conditions for 11 days; different developmental stages of spike formation (5 to 50 mm); and different developmental stages of spike and seed formation after the spikes had emerged from the flag leaf (visible).

For Library 6, seeds were germinated for 7 days and cold-treated as described for Library 2, except that cold treatments were performed for short time points (1, 3 and 6 hr) in the light or in the dark. Crown sections (1 cm) and green leaf tissues were harvested individually for each time point and for both exposure conditions.

For SSH libraries TaLT2 to TaLT7, plants were germinated as described for Library 2 except that the light intensity was 275 μm m^-2 ^s^-1 ^and the cold treatment was performed at 2°C for 1, 21 or 49 days. Crown sections (1 cm) were harvested individually for each time point.

#### RNA purification and cDNA synthesis

For Libraries 2 and 3, total RNA was isolated using the phenol method [53] except that the heating step at 60°C was omitted, whereas the TRI Reagent method (Sigma) was used for Libraries 4 to 6 and TRIzol (Life Technologies) was used for the TaLT libraries. For Libraries 2 to 6, poly(A)^+ ^RNA was purified from the total RNA samples using two cycles of an oligo(dT)-cellulose affinity batch-enrichment procedure [53] whereas PolyA Pure (Ambion) was used for the TaLT libraries. Total RNAs were subsequently used for cDNA synthesis. For all libraries, cDNA synthesis was initiated with a *Not*I primer-adaptor (GCGGCCGCCCT_15_) using the 'SuperScript™ Plasmid System with Gateway Technology for cDNA Synthesis and Cloning' kit (Invitrogen). For Libraries 3 to 6, methylated dCTP was added to the first strand reaction mix to prevent cleavage by the NotI restriction enzyme used for directional cloning. For Library 6, the 'GeneRacer' kit (Invitrogen) was used prior to first strand synthesis to dephosphorylate truncated and non-mRNAs, remove the 5' cap structure from intact mRNA, and ligate the gene racer RNA oligo 5'-CGACUGGAGCACGAGGACACUGACAUGGACUGAAGGAGUAGAAA-3'. The precipitation steps in the kit were replaced by the RNeasy Mini Protocol for RNA Cleanup (QIAGEN). For this library, the second strand cDNA was synthesized using *Pfx *DNA polymerase (Invitrogen) and the primer 5'-CGACTGGAGCACGAGGACACTGA-3' homologous to the RNA oligo. The 'SuperScript™ Plasmid System with Gateway Technology for cDNA Synthesis and Cloning' kit (Invitrogen) was used for the remaining steps of the construction of Libraries 2 to 6 except that the precipitation steps without yeast carrier tRNA were replaced by the QIAquick PCR purification procedure (QIAGEN). For the TaLT2, 3, 6 and 7 libraries, the Nitro-pyrrole anchored oligo-dT priming technique was used [54]. For TaLT4 and TaLT5 libraries, the SMART cDNA (Clontech) priming kit was used.

#### Suppression Subtractive Hybridization

For the TaLT libraries, SSH was performed on the RNAs isolated from crowns. For the TaLT2 library, RNA from CI14106 cold-acclimated for 1 day was used as tester RNA and subtracted by SSH against the driver RNA from cv Norstar cold-acclimated for 21 and 49 days (equal amounts of cDNAs were pooled together before subtraction). For TaLT3, 21 and 49-day cold-acclimated CI14106 was subtracted against cv Norstar cold-acclimated for 1 day. For TaLT4, 1 day cold-acclimated PI178383 was subtracted against 21 and 49 days cold-acclimated cv Norstar. For TaLT5, 21 and 49 days cold-acclimated PI178383 was subtracted against 1 day cold-acclimated Norstar. For TaLT6, 1 day cold-acclimated CI14106 was subtracted against non-acclimated CI14106. For TaLT7, 21 and 49 days cold-acclimated CI14106 was subtracted against non-acclimated CI14106.

#### Cloning into vectors

For Libraries 2 to 6, a *Sal*I adaptor (GTCGACCCACGCGTCCG) was ligated to the 5' end of the cDNAs synthesized with the *Not*I primer-adaptor to allow for directional cloning. The first two (for Libraries 3 to 5) or five (for Libraries 2 and 6) fractions eluting from size fractionation column chromatography and containing cDNAs larger than 0.5 kb were pooled for ligation with the vector. About 15 ng of *Sal*I-*Not*I-digested cDNAs was ligated with 50 ng of the pCMV.SPORT6 vector, which contains the attB1 and attB2 site-specific recombination sites flanking the multiple cloning sites. Therefore, clones isolated from these libraries can be rapidly transferred into Gateway™ destination vectors using site-specific recombination (Invitrogen). The libraries were then transformed into ElectroMAX™ DH10B cells (Invitrogen) for Library 2 or ElectroTen-Blue™ cells (Stratagene) for Libraries 3 to 6. For TaLT libraries, the PCR-amplified products of SSH were non-directionally cloned into the pGEM-T vector and transformed into DH5α cells.

### Assessment of library quality and selection of clones for sequencing

Around 6.0 × 10^6 ^primary clones were obtained for Libraries 2 to 6. To determine the average cDNA size, 96 clones were randomly chosen from different libraries and the plasmids digested and characterized on agarose gels. Average insert sizes were estimated at 1300 bp (Library 2: 14% of inserts below 750 bp, 59% between 750 and 1500 bp, and 27% above 1500 bp), 1560 bp (Library 3: 10% below 750 bp, 44% between 750 and 1500 bp, and 46% above 1500 bp), and 1100 bp (Library 6: 17% below 750 bp, 68% between 750 and 1500 bp, and 15% above 1500 bp). Since all libraries contain an average of 6 million different clones, this collection represents an important resource to isolate full length clones for which only truncated cDNAs are available. To reduce the number of ESTs representing highly expressed genes, Libraries 2 to 6 were hybridized to ^32^P-labelled cDNAs from non-acclimated plants. Colonies showing with the weakest hybridization signals were picked for sequencing.

### Bioinformatics

#### Trimming high quality sequences

Sequence tracefiles were obtained from the FGAS project (110,544 ESTs) and from the NSF (82,332 ESTs; [55]) and DuPont (154,171 ESTs) collections. The latter two collections comprise EST sequences derived from many cDNA libraries prepared from various wheat RNA sources. All sequences were processed as follows. Quality score sequences were obtained from tracefiles using PHRED [56,57]. Only sequences with mean Q≥20 were retained. Poly(A) or poly(T) regions with length = 14 (± 2 errors) were trimmed and all sequences containing more than one poly(A) and/or poly(T) sequences were flagged as putative chimeras. SeqClean3 with generic Univec DB as well as Lucy4 (using pCMV.SPORT6 and pBlueScript II splice sites) were used with the default settings in an iterative manner. This recursive approach proved more efficient in removing vector and linker sequences, and low quality regions than using either one only once. All resulting high quality sequences were then re-checked for low-complexity and all sequences containing more that 50% repeats were rejected. A repeat was defined as a minimum word size of 4 identical bases with a maximum of 1 error. RepeatMasker2 was used with Repeat DB to mask regions that could eventually bias the assembly. All information pertaining to library details, sequences and data quality scores were stored in a mySQL database. After filtering, 269,562 cleaned ESTs were retained for assembly (73,521 ESTs from FGAS, 68,886 ESTs from NSF and 127,155 ESTs from DuPont).

#### Clustering, assembly and annotation

**Figure 4:**
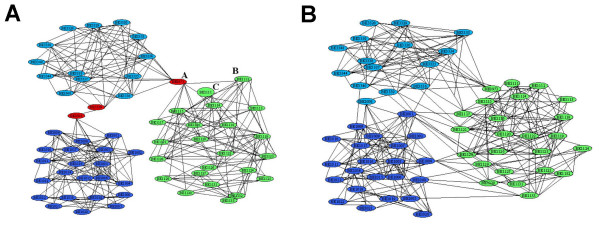
**Breaking strategy of large clusters**. A breaking strategy was used to reduce the size of large clusters. Each sequence in a cluster was BLASTed against the others and e-values were used to build an adjacency matrix (see Materials and Methods). For example, an e-100 value will result in a distance of 1 cm between two sequences. Only values below e-25 were used for graphical display. GRAPH9 was used to flag bridges (articulation points where an EST links two potential sub-clusters) and manually split a cluster into distinct sub-clusters. **A) **Example of a cluster region where specific ESTs (in red) can be manually transferred to sub-clusters (based on the smallest e-value). **B) **Example of a cluster region that could not be broken into sub-clusters due to the complex interrelations between ESTs.

Clustering was performed to reduce the redundancy of the dataset and increase the overall quality of the derived consensus sequences. When a small set of sequences (FGAS 73,521 quality-filtered sequences) was used, the clustering performed well through TGICL and d2_cluster. However, when the NSF and DuPont data (196,041 sequences) were added, aberrant large clusters were obtained. This is presumably due to undetected chimeras, multi-domain proteins and the transitive closure technique applied by these applications. These large clusters (38 k sequences for TGICL and 25 k for d2_cluster) contained many unrelated sequences and were difficult to assemble, yielding many incongruent and low quality contigs. To avoid such artifacts, a cluster breaking strategy was used. First, all sequences that could be contained in other ESTs were removed, thereby reducing the dataset to parent sequences. These sequences were then BLASTed against themselves and results were parsed to extract the e-values in order to build an adjacency matrix. The distance (d) between the sequences was calculated based on the level of similarity established using BLAST e-value where d = 100/-log (e-value). Two parent sequences were considered to be part of the same cluster when the BLASTN identity result between them was greater than or equal to 96%. GRAPH9 was used to flag bridges (articulation points where the removal of an EST breaks the link between sub-clusters) and manually split the large graph into distinct smaller sub-graphs. Other suspicious clusters that were not automatically detected were manually investigated and split when required (Figure 4a). Child ESTs, removed in the first stage were then incorporated into the cluster containing the parent sequence. For example, the largest cluster was broken down using the approach described above and yielded 250 sub-clusters, with the largest being of 6 k sequences (Figure 4b). TGICL and d2_cluster results were compared using randomly chosen clusters that were re-assembled using either clustering tools. It was observed that TGICL had a higher tendency of joining similar genes and falsely splitting sequences from the same gene, thus indicating that d2_cluster was a more reliable clustering tool in our case.

Both CAP3 [58] and PHRAP were tested to assemble the sequences. CAP3 was used on TGICL results using the settings that appeared satisfactory when assembling barley EST sequences [7] while PHRAP was used to assemble d2_cluster results using the default parameters. The first method generated ~32 k contigs while the latter produced over 50 k contigs. The first approach gave results more consistent with the Unigene and TIGR Wheat Gene Index assembly data with respect to contig number, suggesting that PHRAP was less appropriate for assembly of the large dataset used in this study. The total number of singletons and singlets in both cases was similar; 39 k for PHRAP (14% of all ESTs) vs. 42 k for CAP3 (15.5% of all ESTs) and the percentage was close to that found in TIGR (13.3% of all ESTs). Singletons are defined as unique sequences that could not be assembled in a cluster whereas singlets are unique sequences that were assembled in a cluster but could not be assembled in a contig. Based on the TGICL and d2_cluster comparison and on the number of contigs obtained with CAP3 and PHRAP, we chose d2_cluster and CAP3 as the clustering and assembly tools for this project.

We used different annotation tools to increase the number of annotated sequences. The unique assembled sequences produced in our study were annotated after translation using prot4EST and then BLASTed (BLASTX) against a GO-annotated database. All the sequences that did not show sufficient similarity to be functionally classified with this method were investigated with AutoFact where sequences are BLASTed against other complementary databases (ex. PFAM, KEGG, Ribosomal Sequences database) having GO details.

### Digital expression analysis

The relative abundance (digital expression) of FGAS ESTs was analysed as follows: 1) among the contigs containing EST sequences present in both the FGAS dataset and NSF-DuPont dataset, abundance was expressed as a ratio of FGAS ESTs (without SSH ESTs) to NSF-DuPont ESTs, after correction for the size (total number of ESTs) in each dataset; 2) contigs that contained only FGAS ESTs were analyzed separately; 3) SSH EST abundance was compared between similar SSH libraries to determine if common ESTs can be identified; and 4) unique SSH contigs were identified as these could represent new genes expressed during cold acclimation.

### Identification of homologous genes regulated by stress in *Arabidopsis*

The 2,637 putative wheat stress-regulated genes identified in our study were BLASTed (TBLASTX) against the *Arabidopsis *proteins TAIR database [12] using a cut-off e-value of e-25. The Protein ID of the homologous *Arabidopsis *proteins were used to identify those that are represented on the Affymetrix ATH1 genome array and the MWG Biotech 25 k 50-mer oligonucleotide array. The cold- and drought-regulated genes were then identified from the available published data [14,15].

## Authors' contributions

MH, FS, PG, AL and WLC conceived the study and participated in its design and coordination. MH carried out the analyses of the EST datasets and drafted the manuscript. MH, MB and AB carried out the bioinformatics analyses. FO and FS participated in the drafting and editing of the manuscript. JD constructed Libraries 2 to 6. AM, AD and PG prepared the clones from Libraries 2 to 6 for sequencing. AL constructed libraries TaLT2 to TaLT6 and prepared the clones for sequencing. ML, LMcC and WLC carried out the sequencing reactions, the bioinformatics analyses of the FGAS dataset, and submitted the data to Genbank. All authors read and approved the final manuscript.

## Supplementary Material

Additional File 1 **Contigs containing ESTs that are over- or under-represented at least two-fold in the FGAS dataset compared to the NSF/DuPont dataset**. SSH ESTs are not part of this analysis. The contigs containing ESTs over-represented at least 5-fold in FGAS were analyzed by TBLASTX against the *Arabidopsis *TAIR database to find homologues (e-25 cut-off). For those that are represented on the Affymetrix and/or MGW microarrays, the expression data with respect to cold or drought regulation was obtained. U, up-regulated; D, down-regulated. Click here for file

Additional File 2 **Contigs containing at least three ESTs that are present only in the FGAS dataset**. SSH ESTs are not part of this analysis. The contigs were analyzed by TBLASTX against the *Arabidopsis *TAIR database to find homologues (e-25 cut-off). For those that are represented on the Affymetrix and/or MGW microarrays, the expression data with respect to cold or drought regulation was obtained. U, up-regulated; D, down-regulated. Click here for file

Additional File 3 Contigs containing at least three ESTs that are present only in the TaLT libraries of the FGAS dataset. Click here for file

## Figures and Tables

**Table 1:** Summary of tissues used for the different cDNA libraries generated for the FGAS EST sequencing project.

**Library**	Growth conditions*	**Tissues**	**High quality EST sequences**
Library 2	Control plants; Plants cold acclimated for 1, 23 and 53 days	leaves and crowns	25,240
Library 3	Control plants; Plants cold acclimated for 1, 23 and 53 days; Plants salt stressed for 0.5, 3 and 6 hours	roots	11,382
Library 4	Plants dehydrated on the bench (4 time points) and in a growth chamber (4 time points)	leaves and crowns	2,838
Library 5	Various vernalization and developmental stages through spike formation.	crowns and flowers	6,668
Library 6	Control plants; Plants cold acclimated for short time points (1, 3 and 6 hours) under light or dark conditions	leaves and crowns	7,904
TaLT2	SSH library: Tester: cv. CI14106 cold acclimated for 1 day; Driver: cv Norstar cold acclimated for 21 and 49 days	crowns	2,271
TaLT3	SSH library: Tester: cv. CI14106 cold acclimated for 21 and 49 days; Driver: cv Norstar cold acclimated for 1 day	crowns	1,832
TaLT4	SSH library: Tester: cv. PI178383 cold acclimated for 1 day; Driver: cv Norstar cold acclimated for 21 and 49 days	crowns	2,716
TaLT5	SSH library: Tester: cv. PI178383 cold acclimated for 21 and 49 days; Driver: cv Norstar cold acclimated for 1 day	crowns	2,784
TaLT6	SSH library: Tester: cv. CI14106 cold acclimated for 1 day; Driver: non-acclimated cv. CI14106	crowns	4,961
TaLT7	SSH library: Tester: cv. CI14106 cold acclimated for 21 and 49 days; Driver: non-acclimated cv. CI14106	crowns	4,925

* Libraries 2 to 6 were constructed from wheat cv Norstar.

**Table 2:** Homology search of FGAS contigs. As a first step, the 11,225 FGAS unique sequences were analyzed using the wheat-filtered db_est (NCBI release 2.2.12, Aug-07-2005). The non-homologous transcripts were then analyzed against the wheat protein database to subtract protein homologs. The remaining transcripts were then analyzed in the same manner against the *Arabidopsis *and rice databases and finally against the nr database. The complete homology search was performed at e-25 and e-05 cut-offs. The numbers indicate the number of genes that do not show any homology at the indicated e-value cut-off.

		**e-25**	**e-05**
**Wheat**	BLASTN db_est	2304	1581
	
	TBLASTX	2243	1470

***Arabidopsis***	BLASTN db_est	2238	1470
	
	TBLASTX	1985	1102

**Rice**	BLASTN db_est	1845	987
	
	TBLASTX	1674	855

**nr db_est**	BLASTN	1623	795

**Table 3:** Contigs containing ESTs that are over or under-represented in the FGAS dataset relative to the NSF-DuPont dataset.

**Fold increase/decrease**	**Over-represented ESTs**	**Under-represented ESTs**	**Total**	**Percent of total contigs (31,772)**
20	61	5	66	0.2
10	533	22	555	1.7
5	1959	136	2095	6.6
3	5569	489	6052	19
2	6794	1047	7841	24.7

**Table 4:** Contigs containing ESTs that are over-represented over 20-fold in the FGAS dataset.

**Contig name**	**Annotation**	**Fold representation (FGAS/NSF-DuPont)**	**Reference**
CL91Contig4	No Gene Ontology Hit (Wcor413, manual annotation)	163.30	[59]
CL206Contig4	Low molecular mass early light-inducible protein HV90, chloroplast precursor (ELIP)	94.35	[60]
CL386Contig5	Chitinase (EC 3.2.1.14)	68.94	[31]
CL1959Contig1	Legumin-like protein	68.94	[61]
CL117Contig7	No Gene Ontology Hit (Lea/Rab, manual annotation)	61.69	[62,63]
CL10Contig25	Defensin precursor	54.43	[64]
CL347Contig1	COR39 (WCS120 homolog, manual annotation)	52.61	[65]
CL158Contig8	Putative 1-aminocyclopropane-1-carboxylate oxidase	47.17	
CL386Contig1	Chitinase 1	43.54	[31]
CL347Contig2	Cold shock protein CS66 (Wcs120 homolog, manual annotation)	43.54	[65]
CL756Contig2	Hypothetical protein 259I16.2b (LEA homolog, manual annotation)	43.54	[66]
CL1620Contig2	No Gene Ontology Hit	32.66	
CL411Contig1	Putative phytosulfokine receptor (Wheat Ice recristallization inhibitor, manual annotation)	32.66	[32]
CL349Contig4	Ferredoxin-NADP(H) oxidoreductase	32.66	[45]
CL1918Contig1	Glycosyltransferase	32.66	[16]
CL2Contig21	Hypothetical protein (Fragment) (Cab binding protein, manual annotation)	32.66	Genbank U73218
CL756Contig3	No Gene Ontology Hit	29.03	
CL2Contig9	Hypothetical protein (Fragment) (Cab binding protein, manual annotation)	29.03	Genbank U73218
CL3270Contig2	No Gene Ontology Hit	29.03	
CL28Contig11	Extracellular invertase (EC 3.2.1.26)	29.03	[67]
CL650Contig2	Cold acclimation protein WCS19	26.30	[68]
CL1442Contig1	Putative major facilitator superfamily antiporter	25.40	
CL1698Contig3	No Gene Ontology Hit	25.40	
CL704Contig4	Legumin-like protein	25.40	[61]
CL4965Contig1	Hypothetical protein P0508B05.10	25.40	
CL4930Contig1	ATP-dependent RNA helicase	25.40	[69]
CL411Contig4	No Gene Ontology Hit (Wheat Ice recristallization inhibitor, manual annotation)	25.40	[32]
CL2910Contig2	CONSTANS-like protein CO6	25.40	
CL117Contig3	No Gene Ontology Hit (Lea/Rab)	25.40	[63]
CL4699Contig1	Cytochrome P450	25.40	
CL4567Contig1	No Gene Ontology Hit	25.40	
CL1631Contig3	Beta-1,3-glucanase	25.40	[33]
CL411Contig3	Putative phytosulfokine receptor (Wheat Ice recristallization inhibitor, manual annotation)	25.40	[32]
CL91Contig8	No Gene Ontology Hit (COR413, manual annotation)	25.40	[59]
CL2020Contig1	No Gene Ontology Hit	23.58	
CL1106Contig2	Putative cytochrome c oxidoreductase	23.58	[70]
CL280Contig5	No Gene Ontology Hit (blt14, manual annotation)	23.58	[71]
CL1911Contig2	Putative cysteine proteinase inhibitor	21.77	[72]
CL3036Contig1	No Gene Ontology Hit hypothetical protein (OSJNBa0062C05.24, manual annotation),	21.77	
CL171Contig6	No Gene Ontology Hit	21.77	
CL202Contig14	No Gene Ontology Hit	21.77	
CL2484Contig2	No Gene Ontology Hit (putative F-Box family, manual annotation)	21.77	
CL3205Contig2	Hypothetical protein At2g43940	21.77	
CL117Contig2	No Gene Ontology Hit (Lea/Rab)	21.77	[63]
CL2663Contig3	Serine carboxypeptidase I precursor (EC 3.4.16.5) (Carboxypeptidase C) (CP-MI)	21.77	
CL4989Contig1	No Gene Ontology Hit	21.77	
CL437Contig6	Putative family II lipase EXL4	21.77	
CL2012Contig3	CIPK-like protein 1 (EC 2.7.1.37) (OsCK1)	21.77	[73]
CL1442Contig3	Putative major facilitator superfamily antiporter (sugar transporter family, manual annotation)	21.77	
CL3511Contig1	Similarity to receptor protein kinase (leucine rich protein similar to TIR1, manual annotation)	21.77	[74]
CL861Contig1	No Gene Ontology Hit	21.77	
CL4814Contig1	Putative cinnamyl alcohol dehydrogenase	21.77	
CL2Contig49	Chlorophyll a/b-binding protein WCAB precursor	21.77	Genbank U73218
CL4798Contig1	No Gene Ontology Hit	21.77	
CL1740Contig2	Hypothetical protein OSJNBa0086E02.13 (Hypothetical protein P0419C04.2) (putative haloacid dehalogenase-like hydrolase, manual annotation)	21.77	
CL4476Contig1	No Gene Ontology Hit (phosphate induced protein, manual annotation)	21.77	
CL4337Contig1	Putative o-methyltransferase	21.77	[75]
CL2623Contig1	No Gene Ontology Hit (lumenal protein subunit of photosystem II, manual annotation)	21.77	
CL3656Contig2	Barwin	21.77	
CL671Contig1	No Gene Ontology Hit	21.77	
CL878Contig3	Putative pollen allergen Jun o 4	21.77	
CL26Contig8	No Gene Ontology Hit	0.050	
CL350Contig1	Photosystem II reaction center Z protein	0.040	
CL185Contig1	Chloroplast 50S ribosomal protein L14	0.037	
CL120Contig2	Lipid transfer protein 1 precursor	0.030	
CL144Contig2	Alpha amylase inhibitor protein	0.026	

**Table 5:** Contigs containing at least 5 ESTs that are unique to the FGAS dataset.

**Contig name**	**Annotation**	**Number of ESTs**	**Reference**
CL1638Contig1	No Gene Ontology Hit (no homology)	24	[76]
CL1293Contig2	Wheat cold acclimation protein Wcor80 (Wcs120 homolog, manual annotation)	19	[65]
CL386Contig3	Chitinase 1	18	[31]
CL347Contig3	Cold acclimation protein WCS120 (manual annotation)	17	[65]
CL2466Contig1	Putative heat shock protein (E. Coli contaminant, manual annotation)	16	
CL3394Contig1	Nitrogen regulation protein NR(II) (EC 2.7.3.-) (E. coli contaminant, manual annotation)	12	
CL7Contig23	Aquaporin PIP1		[77]
CL40Contig14	Chitinase IV	11	[31]
CL650Contig3	Chloroplast-targeted COR protein (Wcor14c, manual annotation)		[76]
CL1239Contig3	Putative LMW heat shock protein	10	
CL2570Contig1	Hypothetical protein OJ1015F07.4		
CL125Contig7	O-methyltransferase	9	[75]
CL206Contig11	Low molecular mass early light-inducible protein HV90, chloroplast precursor (ELIP)		[60]
CL3635Contig1	No Gene Ontology Hit		
CL4047Contig1	ABA responsive protein mRNA (manual annotation)		[78]
CL52Contig12	No Gene Ontology Hit		
CL52Contig13	No Gene Ontology Hit		
CL619Contig5	WSI76 protein induced by water stress (galactinol synthase, manual annotation)		[79]
CL1228Contig3	Leaf senescence protein-like	8	
CL1293Contig1	Dehydrin (Wcs120 homolog, manual annotation)		[65]
CL2543Contig2	No Gene Ontology Hit		
CL400Contig4	Cysteine protease		[80]
CL4107Contig1	No Gene Ontology Hit		
CL4776Contig1	Probable arylsulfatase activating protein aslB (E. coli contaminant, manual annotation)		
CL1051Contig5	C repeat-binding factor 2	7	[81]
CL2204Contig1	No Gene Ontology Hit (Wheat Ice recristallization inhibitor, manual annotation)		[32]
CL3474Contig1	No Gene Ontology Hit		
CL3792Contig1	No Gene Ontology Hit		
CL4454Contig1	No Gene Ontology Hit		
CL5468Contig1	Ubiquinone/menaquinone biosynthesis methyltransferase ubiE (EC 2.1.1.-) (E. coli contaminant, manual annotation)		
CL833Contig4	Putative EREBP-like protein (putative AP2 domain transcription factor, manual annotation)		
CL1318Contig2	S-like Rnase	6	[82]
CL1368Contig4	Beta-expansin		
CL17Contig3	Type 1 non-specific lipid transfer protein precursor (Fragment)		[83]
CL20Contig27	No Gene Ontology Hit		
CL2425Contig2	Putative lectin		[84]
CL280Contig2	Low temperature responsive barley gene blt14 (manual annotation)		[62]
CL280Contig4	Cold regulated protein pao29 (similar to blt14 manual annotation)		[62]
CL2910Contig1	CONSTANS-like protein CO6		
CL3212Contig2	No Gene Ontology Hit		
CL3324Contig2	RING zinc finger protein-like		
CL3647Contig2	No Gene Ontology Hit		
CL3778Contig2	Putative phenylalanyl-tRNA synthetase alpha chain		
CL4292Contig1	C2H2 Zinc finger protein (manual annotation)		
CL4895Contig1	No Gene Ontology Hit		
CL5228Contig1	Putative inositol-(1,4,5) trisphosphate 3-kinase		
CL5712Contig1	Putative ABCF-type protein (anthocyanin transport)		
CL5985Contig1	Hypothetical protein P0508B05.10		
CL6056Contig1	Putative calcium binding EF-hand protein (caleosin: lipid body trafficking, manual annotation)		
CL6257Contig1	No Gene Ontology Hit		
CL6493Contig1	No Gene Ontology Hit		
CL861Contig2	No Gene Ontology Hit		
CL1051Contig2	C repeat-binding factor 2	5	[81]
CL1182Contig3	OSJNBa0043A12.18 protein (putative transcription factor)		
CL1279Contig2	Isoflavone reductase homolog (EC 1.3.1.-)		
CL1366Contig3	Putative UDP-glucose: flavonoid 7-O-glucosyltransferase		
CL206Contig6	High molecular mass early light-inducible protein HV58, chloroplast precursor (ELIP)		[60]
CL3647Contig1	No Gene Ontology Hit		
CL4058Contig1	Myb-related protein Hv33		
CL411Contig7	No Gene Ontology Hit (Wheat Ice recristallization inhibitor, manual annotation)		[32]
CL4350Contig2	Similarity to protein kinase		GenBank AY738149
CL4537Contig1	Putative ACT domain-containing protein		
CL4642Contig1	Chitinase 1		[31]
CL4666Contig1	Farnesylated protein 1		[85]
CL4825Contig1	Hypothetical protein P0473D02.6 (Hypothetical protein OJ1368_G08.21)		
CL6137Contig1	No Gene Ontology Hit		
CL6258Contig1	Putative sodium-dicarboxylate cotransporter		
CL6567Contig1	Putative arabinogalactan protein		
CL6634Contig1	No Gene Ontology Hit		
CL6741Contig1	Putative b-keto acyl reductase (fatty acid elongase, waxes biosyntheisis)		
CL6821Contig1	Putative strictosidine synthase (alkaloid biosynthesis)		
CL7090Contig1	No Gene Ontology Hit		
CL721Contig3	No Gene Ontology Hit		
CL7241Contig1	No Gene Ontology Hit		
CL7243Contig1	No Gene Ontology Hit		
CL7272Contig1	Early light-inducible protein		[60]
CL7415Contig1	No Gene Ontology Hit		
CL7455Contig1	ABC1 family protein-like		
CL754Contig3	Chitinase 3		[31]
CL7581Contig1	Aspartate transaminase, mitochondrial		
CL7608Contig1	Putative aspartic proteinase nepenthesin I		
CL7617Contig1	No Gene Ontology Hit (barley Blt14 homolog, manual annotation)		[62]
CL7686Contig1	No Gene Ontology Hit		
CL7701Contig1	Putative FH protein interacting protein FIP2 (potassium channel tetramerization)		
CL7785Contig1	No Gene Ontology Hit		
CL7794Contig1	No Gene Ontology Hit		
CL807Contig3	Putative diphosphonucleotide phosphatase (calcineurin-like phosphoesterase)		
CL861Contig5	No Gene Ontology Hit		
CL963Contig4	OSJNBb0013O03.11 protein (bHLH transcription factor, manual annotation)		

**Table 6:** Annotated contigs that are unique to the TaLT libraries (SSH).

**Contig name**	**Annotation**	**Number of ESTs**	**Contigs with similar annotation containing ESTs over-represented in FGAS**
CL1246Contig2	Putative high-affinity potassium transporter	29	
CL1122Contig2	Putative phosphoribosylanthranilate transferase	27	7-fold 7e-53 CL10525Contig1
CL1701Contig1	Potential phospholipid-translocating ATPase	23	
CL1961Contig1	Transcriptional factor B3-like	20	
CL1506Contig2	DHHC-type zinc finger domain-containing protein-like	19	
CL2126Contig1	Putative ACT domain-containing protein	19	
CL622Contig3	50S ribosomal protein L22-like	19	
CL2193Contig1	Putative DEAD/DEAH box RNA helicase protein	17	
CL1038Contig2	Pollen-specific calmodulin-binding protein	16	
CL3163Contig1	ATP synthase protein 9, mitochondrial precursor (EC 3.6.3.14) (Lipid-binding protein)	12	
CL3186Contig1	Putative pollen specific protein (Putative ascorbate oxidase)	12	
CL1986Contig1	Putative dCK/dGK-like deoxyribonucleoside kinase	10	
CL3856Contig1	Protein kinase domain	10	
CL2813Contig3	MKIAA0124 protein (Fragment)	9	
CL4654Contig1	Hypothetical protein OSJNBa0088I06.19	8	
CL4703Contig1	40S ribosomal protein S7	8	
CL4937Contig1	Glyceraldehyde-3-phosphate dehydrogenase (EC 1.2.1.12)	8	
CL1038Contig3	Hypothetical protein AT4g28600	7	
CL4812Contig1	Homeobox transcription factor-like	7	
CL4821Contig1	Agglutinin isolectin 3 precursor (WGA3) (Fragment)	7	
CL4846Contig1	Putative aldo/keto reductase family protein	7	
CL10Contig35	Ribosomal protein L10A	6	
CL4Contig25	Phytochrome B (Fragment)	6	
CL5821Contig1	Putative very-long-chain fatty acid condensing enzyme CUT1	6	7-fold 2e-57 CL5480Contig1
CL5833Contig1	Putative UDP-Gal:betaGlcNAc beta 1,3-galactosyltransferase-I	6	
CL6515Contig1	NBS-LRR disease resistance protein homologue	6	
CL823Contig3	Putative RNA splicing protein	6	
CL1392Contig2	Heat shock factor-binding protein 1	5	
CL4432Contig2	Putative chromomethylase	5	
CL5300Contig2	Hypothetical protein	5	
CL6924Contig1	Beta-expansin (Fragment)	5	7-fold 8e-48 CL235Contig6
CL6960Contig1	Hypothetical protein OSJNBb0027B08.22 (Hypothetical protein OSJNBa0078D06.5)	5	
CL7305Contig1	Agglutinin (CCA)	5	
CL7698Contig1	Putative resistance gene analog PIC27	5	
CL1101Contig4	Putative amino acid transporter	4	
CL1531Contig2	Putative ZIP-like zinc transporter	4	
CL1739Contig3	Putative ethylene-responsive small GTP-binding protein	4	
CL18Contig7	Putative ribosomal protein L5	4	
CL2037Contig3	Protoporphyrin IX Mg-chelatase subunit precursor	4	
CL2221Contig1	Putative Ribosome recycling factor, chloroplast	4	
CL2305Contig1	Eukaryotic translation initiation factor 3 subunit 12 (eIF-3 p25) (eIF3k)	4	
CL3669Contig2	Putative ascorbate oxidase promoter-binding protein AOBP	4	
CL36Contig7	Adenosylhomocysteinase-like protein	4	
CL3840Contig2	Putative aminopropyl transferase	4	
CL6158Contig2	Cytochrome C6, chloroplast-like protein	4	
CL7225Contig1	P0076O17.10 protein	4	
CL732Contig2	OSJNBa0070C17.10 protein	4	
CL7697Contig1	Heat shock factor protein hsf8-like	4	
CL8407Contig1	Aldo/keto reductase family-like protein	4	11-fold 3e-54 CL3996Contig1
CL9543Contig1	Anthranilate N-benzoyltransferase-like protein (AT5g01210/F7J8_190)	4	
CL10751Contig1	Histone H4-like protein	3	7-fold 6e-46 CL9Contig66
CL10863Contig1	Methionine S-methyltransferase (EC 2.1.1.12) (AdoMet:Met S-methyltransferase)	3	
CL11049Contig1	Transferase family	3	
CL12283Contig1	Putative PPR-repeat containing protein	3	
CL12337Contig1	U3 small nucleolar RNA-associated protein 14 (U3 snoRNA-associated protein 14)	3	
CL12711Contig1	Putative lipase/acylhydrolase (Putative anther-specific proline-rich protein)	3	
CL1347Contig2	Omega-3 fatty acid desaturase	3	
CL1402Contig2	Putative VIP2 protein	3	
CL1688Contig3	Putative plastid ribosomal protein L11	3	
CL1Contig342	Protein H2A	3	15-fold 8e-74 CL1Contig113
CL1Contig350	Protein H2A	3	15-fold 2e-47 CL1Contig113
CL1Contig361	60S ribosomal protein L17-1	3	
CL2045Contig1	Cap-binding protein CBP20	3	
CL2470Contig2	Putative inorganic pyrophosphatase	3	7-fold 1e-75 CL2470Contig1
CL2890Contig3	Mak3 protein-like protein	3	7-fold 4e-91 CL2890Contig1
CL3033Contig2	Putative serine/threonine phosphatase	3	
CL3124Contig2	Putative ATP phosphoribosyl transferase	3	
CL4048Contig2	Boron transporter	3	
CL4808Contig2	Putative DNA topoisomerase II	3	
CL617Contig3	Putative calreticulin	3	5-fold 9e-152 CL617Contig1
CL7904Contig1	Hypothetical protein OSJNBb0004M10.19	3	
CL9749Contig1	Putative subtilisin-like proteinase	3	9-fold 3e-20 CL5317Contig1
CL9993Contig1	Hypothetical protein At1g78915	3	
CL4836Contig2	MtN3-like	2	

**Table 7:** Transcription factors that are differentially expressed in the FGAS dataset relative to the NSF-DuPont dataset.

**Transcription factor family**	**over-represented 2 to 5-fold**	**over-represented over 5-fold**	**Contigs unique to FGAS with at least 3 ESTs**	**TOTAL**
AP2 (ex. CBF1,2,3, Aintegumenta)	4	7	9	20
BHLH (Ex. AtMYC2)	5	2	0	7
BZIP (Ex. FD)	5	3	0	8
CCAAT-box transcription factor	2	1	0	3
DEAD/DEAH box helicase	4	4	0	8
F-box protein family	3	0	0	3
FLOWERING LOCUS T	1	0	1	2
GIGANTEA protein	0	1	1	2
Homeodomain Leucine zipper protein (Ex. ABF3 ABF4, ABA response)	2	2	1	5
MADS box transcription factor (Ex. TaVRT1)	2	0	0	2
MYB (Ex. AtMYB2)	14	7	2	23
NAC-domain containing protein (Ex. RD26 dehydration)	11	8	0	19
PHD finger (Ex. pollen development, chromatin-mediated transcription regulation, a variant of Zn-finger)	2	1	0	3
RING finger containing protein (Ex. HOS1 regulating cold response, A variant of Zn finger)	14	4	3	21
SCARECROW gene regulator-like (Ex. Oxidative stress)	3	1	0	4
WD-repeat containing protein	0	1	0	1
WRKY transcription factor (Ex. Drought, oxidative stress and pathogen induced)	14	7	7	28
Zinc finger protein (Ex. CO, Indeterminate-related)	30	11	6	47
Other Transcription factor-like	113	47	14	174
Other DNA-binding protein	143	46	11	200

Total	372	153	55	580
